# A novel multi-target regression framework for time-series prediction of drug efficacy

**DOI:** 10.1038/srep40652

**Published:** 2017-01-18

**Authors:** Haiqing Li, Wei Zhang, Ying Chen, Yumeng Guo, Guo-Zheng Li, Xiaoxin Zhu

**Affiliations:** 1 Department of Control Science and Engineering, Tongji University, Shanghai, 201804, China; 2 Institute of Chinese Materia Medica, China Academy of Chinese Medical Science, Beijing, 100700, China

## Abstract

Excavating from small samples is a challenging pharmacokinetic problem, where statistical methods can be applied. Pharmacokinetic data is special due to the small samples of high dimensionality, which makes it difficult to adopt conventional methods to predict the efficacy of traditional Chinese medicine (TCM) prescription. The main purpose of our study is to obtain some knowledge of the correlation in TCM prescription. Here, a novel method named Multi-target Regression Framework to deal with the problem of efficacy prediction is proposed. We employ the correlation between the values of different time sequences and add predictive targets of previous time as features to predict the value of current time. Several experiments are conducted to test the validity of our method and the results of leave-one-out cross-validation clearly manifest the competitiveness of our framework. Compared with linear regression, artificial neural networks, and partial least squares, support vector regression combined with our framework demonstrates the best performance, and appears to be more suitable for this task.

Predicting drug efficacy with the assistance of machine learning techniques gives guidance to doctors on curing different patients in specific situations. Selecting sensitive drugs in a timely manner is helpful which can prevent the spread of diseases, promote patients’ recovery, enable doctors to use drug rationally and save medical resources. In clinical treatment, individuals may show different sensitivities to the identical drug. Due to the difficulty for doctors to estimate the effect of drugs on patients, they often choose programs for diagnosis and treatment. A period is needed for doctors to determine the effectiveness, and then deciding whether to continue using this medication or switch to another drug. In most cases, this approach can achieve good results. However, sometimes there may be a delay in giving the medication during the best time of treatment.

As a typical medicine, Wuji pill^1^,^2^,^3^,^4^ is prescribed for irritable bowel syndrome^5^,^6^, ulcers, and other gastrointestinal diseases. Wuji pill consists of coptis, evodia fructus and radix paeoniae alba, but according to the variety of ancient medical books^7^, the compatibility proportion of Wuji pills is diverse. In the 2010 edition of Chinese Pharmacopoeia, the compatibility proportion of coptis, evodia rutaecarpa and radix paeoniae is 6:1:6. Studies have shown that with different compatibility proportions, the efficacy is variational. For example, when the compatibility proportion is 1:1:1, Wuji pill is more effective as an analgesic; however, when the proportion is 5:1:1, Wuji pill is better as an anti-inflammatory drug. Therefore, predicting drug efficacy with different compatibility proportions is necessary and meaningful.

Traditional way to measure the efficacy of a drug is achieved by observing the patient’s physical signs and determining the drug concentration in human blood. Van Westen *et al*.^8^ employed proteochemometric models generated from antivirogram data to predict the HIV inhibitor efficacy. Qiu *et al*.^9^ used multiple kernel support vector regression to solve the siRNA efficacy prediction problem. Yamada *et al*.^10^ studied the efficacy prediction of cevimeline in patients with sjögren’s syndrome, and multiple regression is employed to examine the relative contributions of the clinical and immunological factors. According to pharmacokinetic studies, there is a complex process for medicine to take effect. The oral drug is absorbed into the bloodstream through the stomach, so we determine the drug efficacy through the concentration of drugs in the blood. Blood-drug concentration at different time constitutes a time series, then we can infer many pharmacokinetic indicators by recording the changing process of the drug in patient’s blood. In this paper, we predict the drug efficacy by utilizing a blood-drug concentration time series, which is considered as a multi-target problem. Traditional time series prediction method of fitting the time series curve is not suitable for this problem. We employ the proportion of different drug compositions as features for these datasets. In addition, we believe that there might be some correlations between the targets at different time, so we add corresponding targets as assistance to predict the value of the current time.

Recently, many approaches have been proposed to deal with the increasing and challenging multi-target prediction task. Multi-target regression^11^,^12^,^13^,^14^,^15^ which is also known as multi-output, multi-variate^16^,^17^,^18^, or multi-response regression^19^,^20^, aims to predict the value of multiple real-valued target variables simultaneously. Many researchers have studied the multi-target regression problem. Kocev *et al*.^13^ used single- and multi-target regression to model a compound index of vegetation conditions. Burnham *et al*.^21^ applied multi-target regression to infer concentrations of analytes with multi-variate spectral data. Tuia *et al*.^22^, who used multi-output support vector regression to simultaneously estimate the different biophysical parameters from remote sensing images, and it is also used to predict the wind noise intensity of vehicle components^23^. Hanen *et al*.^24^ categorized state-of-the-art multi-target regression methods as transformation methods and algorithm adaptation methods. Problem transformation methods convert the multi-target regression problem into single-target problems, build models for each target, and then concatenate all the predictions. Spyromitros-Xioufis *et al*.^14^ transformed multi-label classification methods to deal with the multi-target regression problem. The are inspired by popular multi-label classification methods, and proposed multi-target regressor stacking and regressor chains for multi-target regression. They used ridge regression^25^, support vector regression machines^26^, regression trees^27^, and stochastic gradient boosting^28^ in their experiments. The researchers believed that approaches based on a single label may be used for multi-target regression by utilizing regression instead of a classification algorithm. Meanwhile, Tsoumakas *et al*.^15^ proposed a new problem transformation method based on the random k-labelsets (RAkEL) method.

Chen *et al*. used latent tree models to analyze Chinese medicine formula data and to reveal the underlying latent structures in predicting drug efficacy prediction^29^. They analyzed the herb prescription data for patients who have a condition known in Chinese medicine as “disharmony between liver and spleen syndrome(DBLS)”. Poon *et al*. introduced a parameter-free algorithm to discover all the possible sets of interacting herbs, which might lead to a good outcome^30^. The efficacy of a traditional Chinese medicine medication derived from the complex interactions of herbs in a formula, is considered as a problem in efficacy prediction.

In this paper, the multi-target regression framework is proposed to deal with the time-series prediction of drug efficacy problem. With a comparative study of Time-Linear Regression (LR), Polynomial Regression (PR)^31^, Support Vector Regression (SVR)^32^,^33^,^34^, Artificial Neural Networks (ANN)^35^,^36^,^37^ and Partial Least Squares Regression (PLSR)^38^,^39^ methods which are applied in a real data set of the drug efficacy of Wuji pill, our framework demonstrates the superiority.

## Materials and Methods

### Datasets and experimental procedure

Datasets are obtained from the Institute of Chinese Materia Medica at the China Academy of Chinese Medical Sciences. The coptis, evodia, and peony extracts (19.3%, 17.7%, 9.37%) come from the China-Japan Friendship Hospital as dried solid powder in vacuo. The mass fractions of each extract are included as follows: in coptis extract, berberine (Ber) 23.03%, palmatine (Pal) 5.52%; in evodia extract, evodiamine (Evo) 0.38%, rutecarpine (Rut) 0.48%; and in peony extract, paeoniflorin (Pae) 13.41%. The compatibility prescription is a mixture of Chinese medicinal herbs, and all experiments used the same batch of extracts.

L_9_(3^4^) orthogonal design is used to study on Wuji pill in this experiment. According to the clinic dose and proportion of the pharmacopoeia of 2010 edition and medical prescription in all dynasties, coptis, evodia and peony are designed to 3 levels, then 9 compatibility prescriptions (1#~9#) are obtained, and the corresponding prescriptions for each herb are designed as comparison (10#~18#) for a total of 18 prescriptions, shown in Table 1.

**Figure 1:**
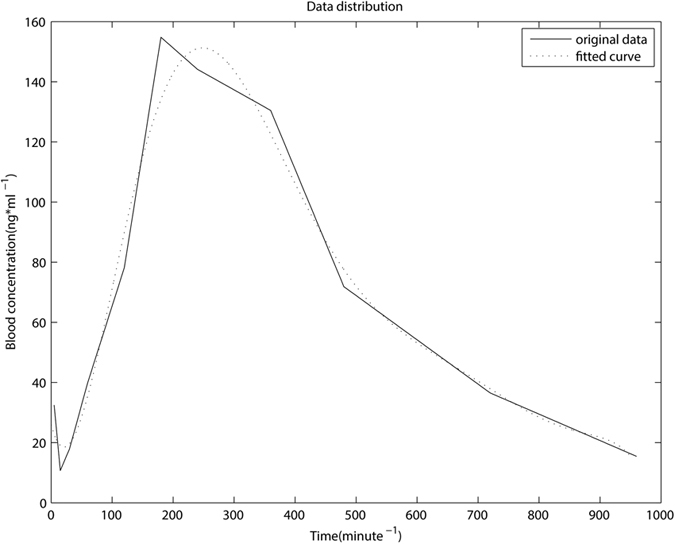
Data distribution of original data and fitted curve. Only part of the Cmax data was chosen as original data in order to show it more clearly. The dashed curve was obtained by polynomial regression, whose order was set to two.

Based on the above orthogonal design, we form 12 groups of prescription compatibility, each is used in 3 experiments, totally producing 36 samples. For each sample, we get the time-blood curve of drug concentration (see Fig. 1) and then we calculate the first order elimination constant (Lambda_z), delay time (Tlag), the time of maximum blood drug concentration (Tmax), the maximum blood drug concentration (Cmax), the area under the blood drug-time curve (AUClast), the area under the curve of zero to infinity time blood drug concentration-time (AUCINF_obs), the apparent volume of distribution (Vz_F_obs), and the plasma elimination rate (Cl_F_obs).

As mentioned above, the time series forecasting mentioned herein is obviously different from general time series prediction. Time series forecasting is traditionally fitted by regression algorithms directly, but in this paper, we predict the blood drug concentration values of corresponding moments. We specifically regard different compatibilities as features, and value of time series as targets, so as to calculate the prediction. Value of time series measured in this experiment is different for various samples, so we normalize this data, and get a total of 9 points which is in common for each process, they are 5 min, 15 min, 30 min, 60 min, 120 min, 180 min, 240 min, 360 min, 480 min. To better understanding the distribution of blood concentration data, we demonstrate it on Fig. 1. The solid line is the curve of original data, and we employed polynomial regression to fit the curve and formed the dashed line, which is expressed with the following formula:


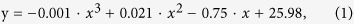


where y means the blood concentration, and x denotes time.

We firstly extract one column as target, then we utilize LR, PR, SVR, ANN, and PLSR to predict a target, lastly, we predict the overall time series with correlation between targets, and the correlation among different time in time series. We then propose a multi-target regression time series prediction framework for this data, and it shows significant improvement of the prediction accuracy.

### Problem definition and algorithm framework

When the medical components are determined, infering the prescription compatibility from blood-drug time series data can be defined as a classification problem. If we consider each medicine component as variable, then this problem is regarded as a regression problem, in which prescription compatibility is inferred by time series data. In reality, we hope every component is changeable, so in this experiment, it can be regarded as a regression problem, in which the time-blood-drug concentration curve can be predicted by varying prescription compatibility. Traditional approach utilizes the regression prediction of every time node and does not consider about the correlation among time nodes. Therefore, we add target of the previous time except the first one to the feature set to improve the prediction precision.

**Figure 2:**
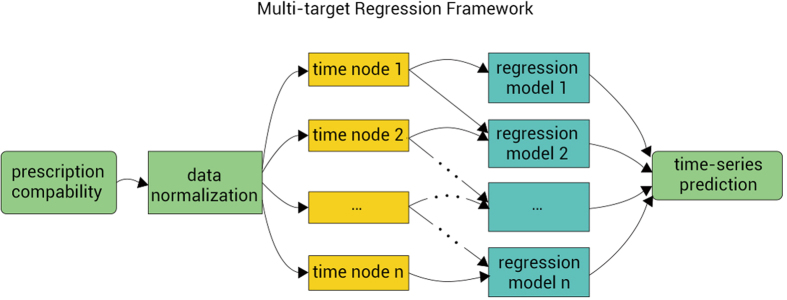
Multi-target Regression Framework. Relation among Time-series data is utilized here to improve the precision of prediction.

Motivated by this idea, we use multi-target regression framework to process the datasets as shown in Fig. 2. <*x*_1_, *x*_2_, *x*_3_> is the input feature set used to predict the time series of blood-drug concentration denoted as <*y*_1_, *y*_2_, *y*_3_, …*y*_*n*_> and 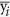 is the predicted value of the ith time node. Considering the correlation between x and y, we add 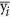 to <*x*_1_, *x*_2_, *x*_3_>, hence we get 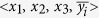, which is used to generate a model and predicted y_*i*+1_. After that, we get 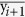, then we add 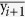 to <*x*_1_, *x*_2_, *x*_3_> which is used to generate a model and predict targets iteratively. In this framework, we add the value of the latest target as one feature to improve the precision of prediction. LR, PR, SVR, ANN and PLSR are used to train the input data and generate models.

According to this framework, we describe the multi-target regression algorithm in Table 2.

## Results

### Assessment of algorithms

The regression accuracy is computed using the learning algorithms described above, which are measured by using the following error measures:

Root mean square error (RMSE) for the *j*th component is defined as


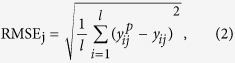


and for the whole, it is


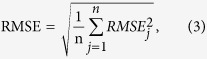


where 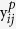 means the *j*th predicted target value of ith example, y_ij_ means the *j*th real target value of ith example, *l* denotes the number of cross validations and n denotes the number of targets, which is 9 in this paper.

Mean absolute error (MAE) for the *j*th component is defined as


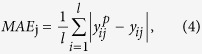


and for the whole, it is


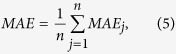


where 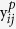, *l* and n have the same meaning as in the definition of RMSE mentioned above.

Mean absolute percentage error (MAPE), for the *j*th component, is defined as


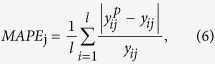


and for the whole, it is


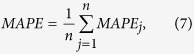


### Results and analysis

Datasets in this experiment are processed by LR, PR, SVR, ANN, and PLSR. During the experiment, the max order of polynomial regression is set to 2, and the number of the hidden layers of ANN is set to 1. When it comes to SVR, the linear kernel is adopted as the kernel function. Leave-one-out is used as the validation method. The results of MAE, MAPE and RMSE are illustrated in Tables 3, 4 and 5.

**Figure 3:**
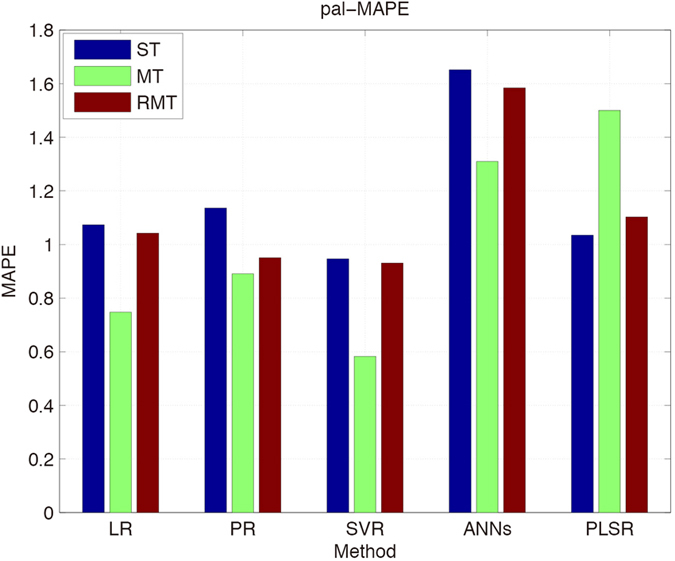
Mean absolute percentage error of pal. Pal data is trained with LR, PR, SVR, ANN, PLS, MT, RMT and leave-one-out cross validation is applied to test the performance.

We then adopt the multi-target (MT) regression framework and calculate the errors for the five components. The random multi-target^15^ is employed as a comparison. For single target regression, the best result of each component is distributed randomly, it is difficult to find a method that satisfied every dataset, so we try to find the relationship between targets to improve the precision of most datasets. Multi-target regression framework could reduce the error and improved the precision for many datasets as shown in Fig. 3. In all methods, SVR combined with multi-target regression framework performs better than the other methods and seems to be more suitable for this task.

Based on the error results of the Tables 3, 4, 5, we calculate the error reduction rate of single-target regression and multi-target regression, as shown in Tables 6, 7, 8, 9, 10, 11, [Table t9], [Table t9], [Table t10], [Table t10], [Table t11]. All the units in the table are percentage, the positive value is the percentage which is improving, and the negative value is the decreasing percentage. most experimental errors decrease with the application of multi-target regression framework for RMSE. Although few results become worse, we even cannot figure it out with the error of single-target regression. When we compare MT with RMT, it is obvious that in few results, RMT is superior to MT, and in most situation, MT performs better. Especially when the basic method is SVR, all the results show that MT is better.

From the improvement percentage of MAPE, it is obvious multi-target regression framework does improve the precision of time series prediction. The result is similar to RMSE’s, most experiments improve the precision a lot, few of them become worse, but the altitude of them can be ignored. For the result of PLS, most of them get worse.

From the improvement percentage of MAE, we draw a conclusion the same as MAPE and RMSE.

All in all, SVR with multi-target regression framework performs better for this data sets and compared with linear regression and other methods, the overall error is the smallest, so we believe multi-target regression framework is suitable for this task.

Experiments in this paper are running on a laptop, the configuration is as follows: GEFORCE GT540M graphics, 1GB video memory, 6 G memory, CPU Intel(R) Core(TM) i5-2410, dominant frequency 2.3 GHz, operating system is 64-bit Windows 7 Ultimate.

## Discussion

The thorough comparative analysis with the most state-of-the-art approaches reveal that methods with our framework achieve satisfying performance. At the same time, SVR with linear kernel is more suitable for these datasets, which are potentially linear, so PR and ANN are hard to treat this linear data set well^34^, especially when the datasets are small. We set the order of PR as three, four or more, results become worse. The number of hidden layers of ANN is also analyzed with bad results, so the three-layer neural network is adopted. When the relationship between targets is considered, the prediction results from most methods are further improved, while SVR obtained the best results among all of the learning algorithms.

Furthermore, we try to add different combinations of targets to the feature, such as the last two targets, three targets, etc. up to all the targets^14^. They perform worse compared to the results that use the last target. Considering the correlation between them, we believe that the last target has the closest correlation with the current target value. When the correlation between two targets is strong, performance of our algorithm is excellent, otherwise when the value of time series changes rapidly, results become worse. Even though, compared with single target regression, our algorithm obtains better results. Besides, in order to guarantee the performance of our algorithm, attribute estimation of datasets is needed for the optimum solution before choosing a suitable kernel function^40^.

## Conclusion

In this paper, we propose a surprisingly simple, useful, and high quality multi-target regression framework, which employs the correlation between targets to improve performance of learning methods, i.e. LR, PR, SVR, and ANN. We apply the proposed methods to the real-world TCM datasets. Experimental results with three evaluation measures, i.e. MAE, RMSE and MAPE demonstrate that methods with our framework outperform other state-of-the-art ones^34^. This framework efficiently predicts the time series value of blood-drug concentration for different prescription compatibility.

Plasma drug concentration might be as an objective criterion of therapeutic, so topic of discovering plasma drug concentration time series is interesting. Our future work is concentrated on improving results with more careful design by the inclusion of more challenging datasets^24^. Additionally, we are exploring how to utilize plasma drug concentration time series datasets to infer the prescription compatibility, which would offer guidance to design a drug with the specific efficacy^2^.

## Additional Information

**How to cite this article**: Li, H. *et al*. A novel multi-target regression framework for time-series prediction of drug efficacy. *Sci. Rep.* **7**, 40652; doi: 10.1038/srep40652 (2017).

**Publisher's note:** Springer Nature remains neutral with regard to jurisdictional claims in published maps and institutional affiliations.

## Supplementary Material

Supplementary Information

## Figures and Tables

**Table 1:** L_9_(3^4^) test schedule of Wuji pill.

Category	Coptis	Evodia	Peony
1#	0.48	0.15	0.23
2#	0.48	0.29	0.47
3#	0.48	0.88	0.93
4#	0.96	0.15	0.47
5#	0.96	0.29	0.93
6#	0.96	0.88	0.23
7#	1.92	0.15	0.93
8#	1.92	0.29	0.23
9#	1.92	0.88	0.47
10#	0.48	0	0
11#	0.96	0	0
12#	1.92	0	0
13#	0	0.15	0
14#	0	0.29	0
15#	0	0.88	0
16#	0	0	0.23
17#	0	0	0.47
18#	0	0	0.93

**Table 2:** The Multi-target Regression Algorithm.

Input: Training dataset Tr(x, y) and test dataset Ts procedure:
1) Train a model L on the training set Tr with the first time node by using regression algorithm and calculated the training error.
2) Add the prediction value of previous target to the feature set, then use regression algorithm to predict the current target and calculate the training error.
3) Perform step 2 iteratively up to and including the last target. Combine all the parameters and run on the test data.
Output: Test error E_0_

**Table 3:** Comparison among the mean absolute errors of each component, processed with LR, PR, SVR, ANN and PLS.

MAE	ber	pal	evo	rut	pae
LR-ST	61.92 ± 25.78	35.90 ± 9.58	21.91 ± 9.23	12.34 ± 7.39	80.94 ± 43.51
LR-MT	55.54 ± 31.17	27.49 ± 14.84	10.39 ± 6.04	10.21 ± 7.01	47.04 ± 30.26
LR-RMT	51.20 ± 32.10	26.86 ± 12.76	20.65 ± 7.32	13.62 ± 8.24	84.09 ± 47.76
PR-ST	67.42 ± 33.70	35.05 ± 8.02	18.30 ± 7.43	14.89 ± 8.79	65.19 ± 33.99
PR-MT	57.79 ± 35.14	27.88 ± 8.99	18.18 ± 6.87	13.57 ± 7.88	53.23 ± 25.74
PR-RMT	61.78 ± 33.33	30.80 ± 8.87	19.57 ± 8.38	14.98 ± 8.84	70.73 ± 39.22
SVR-ST	62.23 ± 28.11	35.90 ± 8.50	20.62 ± 8.89	11.47 ± 6.46	74.47 ± 42.51
SVR-MT	**50.36** ± **29.75**	**24.59** ± **11.33**	**10.18** ± **5.64**	**9.95** ± **8.31**	**44.60** ± **32.17**
SVR-RMT	62.99 ± 26.37	33.11 ± 12.57	19.86 ± 8.00	12.04 ± 6.73	78.71 ± 45.61
ANN-ST	109.6 ± 46.73	59.84 ± 25.89	34.27 ± 13.32	18.01 ± 9.28	106.08 ± 55.64
ANN-MT	95.26 ± 60.89	47.53 ± 13.99	23.82 ± 9.55	17.86 ± 10.72	82.89 ± 60.43
ANN-RMT	88.68 ± 61.26	51.68 ± 22.84	30.28 ± 14.45	18.15 ± 8.00	110.23 ± 51.89
PLS-ST	64.97 ± 37.20	33.32 ± 7.08	21.39 ± 8.63	18.70 ± 9.47	66.24 ± 32.85
PLS-MT	63.09 ± 31.63	43.65 ± 14.13	64.38 ± 27.25	32.04 ± 21.64	73.38 ± 45.61
PLS-RMT	70.04 ± 32.40	33.90 ± 9.77	24.18 ± 10.69	22.02 ± 14.29	74.65 ± 37.64

Here ST denotes Single-target regression, MT denotes Multi-Target regression with last target, RMT denotes Multi-Target regression with random target.

All data here means MAE ± SD, SD is standard deviation.

**Table 4:** Comparison among the mean absolute percentage errors of each component, processed with LR, PR, SVR, ANN and PLS.

MAPE	ber	pal	evo	rut	pae
LR-ST	0.464 ± 0.128	1.073 ± 0.491	1.950 ± 0.581	0.692 ± 0.144	0.878 ± 0.224
LR-MT	0.375 ± 0.062	0.752 ± 0.431	0.800 ± 0.446	0.491 ± 0.111	0.525 ± 0.199
LR-RMT	0.394 ± 0.156	0.895 ± 0.620	1.874 ± 0.688	0.750 ± 0.177	0.926 ± 0.217
PR-ST	0.511 ± 0.159	1.136 ± 0.333	1.383 ± 0.391	0.677 ± 0.132	0.651 ± 0.125
PR-MT	0.383 ± 0.074	0.890 ± 0.374	1.422 ± 0.391	0.633 ± 0.123	0.493 ± 0.146
PR-RMT	0.436 ± 0.103	0.983 ± 0.346	1.512 ± 0.407	0.694 ± 0.136	0.737 ± 0.201
SVR-ST	0.468 ± 0.172	0.947 ± 0.303	1.413 ± 0.356	0.540 ± 0.131	0.711 ± 0.136
SVR-MT	**0.338** ± **0.060**	**0.574** ± **0.217**	**0.721** ± **0.497**	**0.426** ± **0.212**	**0.473** ± **0.250**
SVR-RMT	0.493 ± 0.203	0.889 ± 0.382	1.302 ± 0.331	0.544 ± 0.139	0.820 ± 0.259
ANN-ST	0.872 ± 0.317	1.846 ± 0.746	3.620 ± 1.667	0.895 ± 0.269	1.286 ± 0.391
ANN-MT	0.654 ± 0.490	1.411 ± 0.454	2.265 ± 0.893	0.957 ± 0.258	1.068 ± 0.712
ANN-RMT	0.668 ± 0.404	1.594 ± 0.816	2.573 ± 1.193	1.063 ± 0.247	1.373 ± 0.362
PLS-ST	0.462 ± 0.137	1.035 ± 0.348	1.529 ± 0.402	0.890 ± 0.288	0.701 ± 0.144
PLS-MT	0.471 ± 0.234	1.507 ± 1.136	5.595 ± 2.686	1.602 ± 0.639	0.899 ± 0.767
PLS-RMT	0.523 ± 0.137	1.051 ± 0.361	1.769 ± 0.461	1.039 ± 0.413	0.775 ± 0.239

Here ST denotes Single-target regression, MT denotes Multi-Target regression with last target, RMT denotes Multi-Target regression with random target.

All data here means MAPE ± SD.

**Table 5:** Comparison among the root mean square errors of each component, processed with LR, PR, SVR, ANN and PLS.

RMSE	ber	pal	evo	rut	pae
LR-ST	66.88 ± 27.08	37.46 ± 9.99	22.49 ± 9.31	13.08 ± 7.48	82.05 ± 43.61
LR-MT	60.92 ± 32.56	29.38 ± 14.66	11.07 ± 6.07	11.17 ± 7.09	48.61 ± 30.77
LR-RMT	**55.52 ± 33.68**	28.52 ± 13.55	21.43 ± 7.56	14.39 ± 8.41	85.85 ± 48.09
PR-ST	71.83 ± 34.23	36.63 ± 8.51	18.89 ± 7.58	15.58 ± 8.84	66.50 ± 34.10
PR-MT	63.03 ± 37.46	29.53 ± 9.23	18.78 ± 6.95	14.32 ± 7.92	54.68 ± 25.97
PR-RMT	66.71 ± 34.41	32.46 ± 9.28	20.36 ± 8.66	15.75 ± 8.96	72.10 ± 39.36
SVR-ST	67.09 ± 28.61	37.37 ± 8.91	21.18 ± 8.98	12.22 ± 6.62	75.79 ± 42.53
SVR-MT	55.72 ± 30.95	**26.42** ± **11.41**	**10.83** ± **5.71**	**10.83** ± **8.29**	**45.96** ± **32.78**
SVR-RMT	68.00 ± 26.80	34.98 ± 12.70	20.67 ± 8.31	12.81 ± 6.92	80.33 ± 45.75
ANN-ST	113.71 ± 47.65	61.09 ± 26.01	34.73 ± 13.38	18.80 ± 9.44	107.3 ± 55.69
ANN-MT	99.38 ± 62.11	49.43 ± 14.31	24.48 ± 9.74	18.89 ± 10.79	84.48 ± 60.39
ANN-RMT	92.62 ± 63.76	53.80 ± 23.29	31.19 ± 14.59	19.11 ± 8.26	113.69 ± 53.18
PLS-ST	69.61 ± 38.04	34.91 ± 7.56	22.02 ± 8.77	19.31 ± 9.58	67.66 ± 32.99
PLS-MT	68.47 ± 32.59	45.02 ± 14.38	64.80 ± 27.23	32.64 ± 21.59	74.72 ± 45.71
PLS-RMT	75.07 ± 33.80	35.62 ± 9.92	24.89 ± 10.83	22.65 ± 14.39	76.24 ± 37.99

Here ST denotes Single-target regression, MT denotes Multi-Target regression with last target, RMT denotes Multi-Target regression with random target.

All data here means RMSE ± SD.

**Table 6:** Percentage improved by MT compared with ST in RMSE.

MT-RMSE-imp[a]	LR	PR	SVR	ANN	PLS
ber	8.91	12.25	16.94	12.60	1.64
pal	21.57	19.39	29.30	19.09	−28.98
evo	50.76	0.56	48.86	29.53	−194.3
rut	14.65	8.07	11.36	−0.05	−69.01
pae	40.76	17.78	39.36	21.27	−10.43

^[a]^MT-RMSE-imp = (ST_RMSE-MT_RMSE)/ST_RMSE*100%.

**Table 7:** Percentage improved by MT compared with RMT in RMSE.

RMT-RMSE-imp[a]	LR	PR	SVR	ANN	PLS
ber	−9.73	5.52	18.06	−7.30	8.79
pal	−3.02	9.03	24.47	8.12	−26.39
evo	48.34	7.76	47.61	21.51	−160.35
rut	22.38	9.08	15.46	1.15	−44.11
pae	43.38	24.16	42.79	25.69	1.99

^[a]^RMT-RMSE-imp = (RMT_RMSE-MT_RMSE)/RMT_RMSE*100%.

**Table 8:** Percentage improved by MT compared with ST in MAPE.

MT-MAPE-imp[a]	LR	PR	SVR	ANN	PLS
ber	19.13	24.95	27.66	24.97	−2.07
pal	29.89	2163	39.37	23.57	−45.56
evo	58.96	−2.80	49.00	37.41	−266.01
rut	29.11	6.50	21.10	−0.69	−80.01
pae	40.17	24.20	33.46	16.93	−28.23

^[a]^MT-MAPE-imp = (ST_MAPE-MT_MAPE)/ST_MAPE*100%.

**Table 9 t9:** Percentage improved by MT compared with RMT in MAPE.

RMT-MAPE-imp[a]	LR	PR	SVR	ANN	PLS
ber	4.82	12.16	31.44	2.10	9.94
pal	15.98	9.46	35.43	11.48	−43.39
evo	57.31	5.95	44.62	11.97	−216.28
rut	34.53	8.79	21.69	9.97	−54.19
pae	43.30	33.11	42.32	22.21	−16.00

^[a]^RMT-MAPE-imp = (RMT_MAPE-MT_MAPE)/RMT_MAPE*100%.

**Table 10 t10:** Percentage improved by MT compared with ST in MAE.

MT-MAE-imp[a]	LR	PR	SVR	ANN	PLS
ber	10.31	14.30	19.07	13.08	2.90
pal	23.41	20.44	31.50	20.56	−31.02
evo	52.55	0.62	50.63	30.48	−200.84
rut	17.21	8.91	13.27	0.83	−71.31
pae	41.89	18.35	40.10	21.87	−10.78

^[a]^MT-MAE-imp = (ST_MAE-MT_MAE)/ST_MAE*100%.

**Table 11 t11:** Percentage improved by MT compared with RMT in MAE.

RMT-MAE-imp[a]	LR	PR	SVR	ANN	PLS
ber	−8.48	6.46	20.05	−7.42	9.92
pal	−2.35	9.48	25.73	8.03	−28.76
evo	49.69	7.10	48.74	21.33	−166.25
rut	25.04	9.41	17.36	1.60	−45.50
pae	44.06	24.74	43.34	24.80	1.70

^[a]^RMT-MAE-imp = (RMT_MAE-MT_MAE)/RMT_MAE*100%.
