# Supplementary material for: A novel multi-target regression framework for time-series prediction of drug efficacy
Source: Sci Rep. 2017 Jan 18;7:40652. doi: 10.1038/srep40652 (PMC5241636; doi:10.1038/srep40652)
Supplement: Supplementary Information [file srep40652-s1.pdf]

Electronic Supplementary Material to the Manuscript:

"A novel multi-target regression framework for time-series prediction of drug efficacy"

Haiqing Li, Wei Zhang, Ying Chen, Yumeng Guo, Guo-Zheng Li and Xiaoxin Zhu

MATERIAL AND METHODS p. 1

Intestine perfusion and sampling procedure p. 1

Data used for this study p. 1

RESULTS p. 14

Basic statistics of the original data p. 14

REFERENCES p.15

Supplementary MATERIAL AND METHODS

Intestine perfusion and sampling procedure

Perfusate contained 0.3% glucose, 10% of washed rat outdated red blood cells, 1% bovine serum albumin, 3% dextran T40. Before the experiment, we add 0.2g of dexamethasone and 0.04g of noradrenaline. Referring to Pang's approach[1], After induction of anesthesia (urethane) from a point on the abdomen, two incisions through the abdominal wall were made to the level of the diaphragm. The hepatic and celiac arteries were ligated. The pyloric vein was cannulated with epidural tube for sampling from portal vein, followed by cannulation of the bile duct. The superior mesenteric artery, right renal artery and aorta were isolated. Loose ligatures were placed around the aorta immediately. The right renal artery was then tied close to the right kidney. A bulldog clamp was placed at the right renal artery just at its point of entry to the aorta. A bull dog clamp was placed at the right renalartery just at its point of entry to the aorta, then into the superior mesenteric artery. Perfusion was started at a flow rate of about 5 ml/min and oxygenated with 95% oxygen-5% carbon dioxide at 1 L/min. The thorax was opened quickly and the right atrium was cut to allow perfusate to exit. A catheter was placed through the opening of the right atrium, with its tip advancing to the entry of the hepatic vein and was secured into place. The flow rate for perfusion was increased to the desired flow rate of 7.5 ml/min, The temperature is maintained at 37℃. Perfusate without red blood cells and dextran was used to wash the residual blood, it's used up when the color of liver turn pale yellow.Before experiment, the model would be washed by 300mL of blank perfusate.Samples were taken from this second reservoir before drug administration and at 5, 15, 30, 60, 120,180, 240, 360 and 480 min after the intraduodenal injection.Add acetonitrile into three fold of perfusate. Stirring for 60s, then get supernatant after high speed centrifugation for further analysis.

Data used for this study (Table 1-59)

Blood-drug concentration of paeoniflorin (Table 1-12)

Table 1. Blood-drug concentration of paeoniflorin when the proportion of coptis, evodia and radix paeoniae alba is 3:1:3.

Time(min)	1#(ng/ml)	2#(ng/ml)	3#(ng/ml)
5	38.19	24.93	32.63
15	57.64	44.82	53.64
30	55.84	58.42	65.84
60	81.43	74.61	80.89
120	97.52	77.92	87.80
180	67.05	58.23	53.92
240	45.26	33.23	15.84
360	22.54	22.34	19.86
480	18.23	23.65	13.69

1# here means drug concentration in the blood of No. 1 rat.

Table 2. Blood-drug concentration of paeoniflorin when the proportion of coptis, evodia and radix paeoniae alba is 3:2:6.

Time(min)	1#(ng/ml)	2#(ng/ml)	3#(ng/ml)
5	30.82	26.48	17.34
15	46.02	39.45	28.34
30	74.05	66.23	59.01
60	81.11	75.76	68.29
120	86.78	89.52	90.53
180	108.97	105.34	115.54
240	148.14	136.87	152.96
360	68.92	56.83	78.98
480	29.85	33.57	41.82

1# here means drug concentration in the blood of No. 1 rat.

Table 3. Blood-drug concentration of paeoniflorin when the proportion of coptis, evodia and radix paeoniae alba is 3:6:12.			
Time(min)	1#(ng/ml)	2#(ng/ml)	3#(ng/ml)
5	103.45	127.32	146.08
15	321.86	346.14	302.84
30	480.24	434.85	408.14
60	575.28	547.34	512.34
120	312.47	342.17	342.17
180	356.23	223.80	276.72
240	240.12	235.94	235.94
360	153.23	102.34	82.86
480	103.45	127.32	146.08
1# here means drug concentration in the blood of No. 1 rat.			

Table 4. Blood-drug concentration of paeoniflorin when the proportion of coptis, evodia and radix paeoniae alba is 6:1:6.			
Time(min)	1#(ng/ml)	2#(ng/ml)	3#(ng/ml)
5	23.87	32.75	36.75
15	53.45	76.34	68.89
30	93.45	84.23	90.78
60	108.53	95.91	97.34
120	106.96	116.57	156.32
180	242.06	236.90	275.34
240	215.76	203.13	206.71
360	85.23	76.23	67.10
480	23.87	32.75	36.75
1# here means drug concentration in the blood of No. 1 rat.			

Table 5. Blood-drug concentration of paeoniflorin when the proportion of coptis, evodia and radix paeoniae alba is 6:2:12.			
Time(min)	1#(ng/ml)	2#(ng/ml)	3#(ng/ml)
5	59.64	65.23	72.12
15	132.45	132.45	110.37
30	156.89	168.71	146.23
60	200.08	234.87	226.57
120	301.23	358.97	332.48
180	344.45	261.70	254.34
240	198.46	212.23	187.29
360	151.23	130.45	110.82
480	59.64	65.23	72.12
1# here means drug concentration in the blood of No. 1 rat.			

Table 6. Blood-drug concentration of paeoniflorin when the proportion of coptis, evodia and radix paeoniae alba is 6:6:3.			
Time(min)	1#(ng/ml)	2#(ng/ml)	3#(ng/ml)
5	32.63	29.18	11.24
15	53.64	45.23	36.34
30	65.84	70.82	55.81
60	80.89	79.23	76.09
120	87.80	88.12	83.12
180	84.23	101.34	87.21
240	79.89	106.23	91.23
360	67.27	99.34	86.73
480	32.63	29.18	11.24
1# here means drug concentration in the blood of No. 1 rat.			

Table 7. Blood-drug concentration of paeoniflorin when the proportion of coptis, evodia and radix paeoniae alba is 12:1:12.			
Time(min)	1#(ng/ml)	2#(ng/ml)	3#(ng/ml)
5	116.52	104.87	101.96
15	200.92	180.83	175.81
30	262.81	236.53	229.95
60	527.21	474.49	461.31
120	688.41	619.57	602.36
180	916.72	825.05	802.13
240	512.40	461.16	448.35
360	166.24	149.62	145.46
480	116.52	104.87	101.96
1# here means drug concentration in the blood of No. 1 rat.			

Table 8. Blood-drug concentration of paeoniflorin when the proportion of coptis, evodia and radix paeoniae alba is 12:2:3.			
Time(min)	1#(ng/ml)	2#(ng/ml)	3#(ng/ml)
5	20.12	36.59	27.44
15	33.08	60.14	45.11
30	44.64	81.17	60.88
60	62.79	114.16	85.62
120	118.33	118.33	88.75
180	198.23	151.52	138.64
240	110.23	135.94	96.95
360	74.23	58.34	43.76
480	20.12	36.59	27.44
1# here means drug concentration in the blood of No. 1 rat.			

Table 9. Blood-drug concentration of paeoniflorin when the proportion of coptis, evodia and radix paeoniae alba is 12:6:6.			
Time(min)	1#(ng/ml)	2#(ng/ml)	3#(ng/ml)
5	22.46	24.71	29.20
15	34.70	38.16	45.10
30	33.39	36.73	43.41
60	57.36	63.10	74.57
120	52.95	68.92	82.34
180	84.94	93.43	110.42
240	113.95	125.35	148.14
360	26.05	46.80	58.91
480	22.46	24.71	29.20
1# here means drug concentration in the blood of No. 1 rat.			

Table 10. Blood-drug concentration of paeoniflorin when the proportion of coptis, evodia and radix paeoniae alba is 0:0:3.			
Time(min)	1#(ng/ml)	2#(ng/ml)	3#(ng/ml)
5	18.29	21.75	25.08
15	30.07	35.76	32.31
30	57.08	53.92	52.76
60	59.16	58.53	53.39
120	25.76	35.95	31.34
180	17.97	10.56	14.80
240	11.90	13.24	11.71
360	9.17	9.13	4.00
480	3.42	3.78	2.92
1# here means drug concentration in the blood of No. 1 rat.			

Table 11. Blood-drug concentration of paeoniflorin when the proportion of coptis, evodia and radix paeoniae alba is 0:0:6.			
Time(min)	1#(ng/ml)	2#(ng/ml)	3#(ng/ml)
5	21.98	26.43	33.41
15	42.48	39.48	47.49
30	72.65	77.27	90.23
60	98.76	107.00	112.00
120	69.45	63.94	90.23
180	43.76	35.20	49.95
240	24.68	32.68	30.36
360	19.90	21.93	13.97
480	11.12	11.92	10.23
1# here means drug concentration in the blood of No. 1 rat.			

Table 12. Blood-drug concentration of paeoniflorin when the proportion of coptis, evodia and radix paeoniae alba is 0:0:12.			
Time(min)	1#(ng/ml)	2#(ng/ml)	3#(ng/ml)
5	40.12	50.23	73.04
15	58.78	65.70	101.42
30	132.56	172.10	204.07
60	193.57	229.18	256.17
120	111.51	128.10	138.36
180	82.14	79.28	67.97
240	63.50	57.59	52.48
360	38.97	41.56	41.43
480	24.67	29.13	22.44
1# here means drug concentration in the blood of No. 1 rat.			

Blood-drug concentration of berberine (Table 13-24)

Table 13. Blood-drug concentration of berberine when the proportion of coptis, evodia and radix paeoniae alba is 3:1:3.

Time(min)	1#(ng/ml)	2#(ng/ml)	3#(ng/ml)
5	26.73	35.55	38.73
15	47.50	53.20	50.35
30	101.75	157.14	71.36
60	192.48	259.13	190.23
120	115.00	111.80	95.11
180	88.13	89.25	61.75
240	68.88	108.10	55.07
360	45.20	61.51	48.20
480	38.18	37.79	15.41

1# here means drug concentration in the blood of No. 1 rat.

Table 14. Blood-drug concentration of berberine when the proportion of coptis, evodia and radix paeoniae alba is 3:2:6.

Time(min)	1#(ng/ml)	2#(ng/ml)	3#(ng/ml)
5	29.31	24.28	44.56
15	55.94	79.62	62.52
30	86.98	102.46	128.54
60	310.16	281.96	277.48
120	216.36	221.72	124.93
180	114.30	126.43	78.28
240	80.35	77.26	55.51
360	26.64	34.52	33.87
480	22.90	19.72	23.66

1# here means drug concentration in the blood of No. 1 rat.

Table 15. Blood-drug concentration of berberine when the proportion of coptis, evodia and radix paeoniae alba is 3:6:12.

Time(min)	1#(ng/ml)	2#(ng/ml)	3#(ng/ml)
5	86.09	100.05	67.87
15	235.55	187.90	251.52
30	146.79	162.58	221.81
60	185.73	122.38	164.25
120	118.23	93.83	103.75
180	85.94	58.48	118.72
240	39.60	59.96	57.53
360	36.84	42.09	45.23
480	24.54	31.90	27.54

1# here means drug concentration in the blood of No. 1 rat.

Table 16. Blood-drug concentration of berberine when the proportion of coptis, evodia and radix paeoniae alba is 6:1:6.

Time(min)	1#(ng/ml)	2#(ng/ml)	3#(ng/ml)
5	65.31	75.30	46.06
15	60.97	148.34	115.84
30	314.37	388.53	305.23
60	279.35	247.47	223.70
120	242.08	190.36	188.68
180	132.86	114.03	164.03
240	110.30	83.11	95.46
360	83.19	93.09	61.16
480	52.69	76.55	47.98

1# here means drug concentration in the blood of No. 1 rat.

Table 17. Blood-drug concentration of berberine when the proportion of coptis, evodia and radix paeoniae alba is 6:2:12.

Time(min)	1#(ng/ml)	2#(ng/ml)	3#(ng/ml)
5	65.31	75.30	46.06
15	60.97	148.34	115.84
30	314.37	388.53	305.23
60	279.35	247.47	223.70
120	242.08	190.36	188.68
180	132.86	114.03	164.03
240	110.30	83.11	95.46
360	83.19	93.09	61.16
480	52.69	76.55	47.98

1# here means drug concentration in the blood of No. 1 rat.

Table 18. Blood-drug concentration of berberine when the proportion of coptis, evodia and radix paeoniae alba is 6:6:3.

Time(min)	1#(ng/ml)	2#(ng/ml)	3#(ng/ml)
5	56.03	80.18	60.65
15	101.37	124.91	148.23
30	237.19	276.79	280.89
60	165.40	211.83	217.48
120	132.71	118.59	160.61
180	119.56	97.04	95.92
240	84.11	78.26	84.79
360	55.93	65.22	47.22
480	103.51	75.57	58.57

1# here means drug concentration in the blood of No. 1 rat.

Table 19. Blood-drug concentration of berberine when the proportion of coptis, evodia and radix paeoniae alba is 12:1:12.

Time(min)	1#(ng/ml)	2#(ng/ml)	3#(ng/ml)
5	40.69	82.96	46.18
15	119.79	150.57	167.04
30	154.49	102.08	216.43
60	240.69	182.96	246.18
120	593.01	284.76	351.22
180	380.77	496.89	518.82
240	440.88	356.85	443.58
360	344.50	260.58	339.24
480	202.12	199.31	159.31

1# here means drug concentration in the blood of No. 1 rat.

Table 20. Blood-drug concentration of berberine when the proportion of coptis, evodia and radix paeoniae alba is 12:2:3.

Time(min)	1#(ng/ml)	2#(ng/ml)	3#(ng/ml)
5	91.19	87.45	66.67
15	109.42	154.39	113.41
30	82.32	197.09	263.45
60	356.53	312.47	389.88
120	590.15	558.21	499.32
180	417.00	367.00	501.56
240	342.10	321.16	346.16
360	304.79	225.28	249.31
480	219.85	165.00	195.48

1# here means drug concentration in the blood of No. 1 rat.

Table 21. Blood-drug concentration of berberine when the proportion of coptis, evodia and radix paeoniae alba is 12:6:6.

Time(min)	1#(ng/ml)	2#(ng/ml)	3#(ng/ml)
5	73.77	87.38	112.80
15	254.82	191.88	148.00
30	624.07	371.83	339.96
60	555.12	553.33	499.58
120	416.47	367.84	190.40
180	281.94	307.30	69.43
240	122.62	100.94	106.50
360	125.44	110.59	72.32
480	93.75	73.04	71.03

1# here means drug concentration in the blood of No. 1 rat.

Table 22. Blood-drug concentration of berberine when the proportion of coptis, evodia and radix paeoniae alba is 3:0:0.			
Time(min)	1#(ng/ml)	2#(ng/ml)	3#(ng/ml)
5	52.41	60.40	31.90
15	78.89	78.68	108.17
30	127.34	125.82	153.29
60	338.80	322.21	286.34
120	198.45	133.43	108.43
180	112.34	107.53	56.09
240	74.09	76.79	43.10
360	31.09	31.38	9.18
480	13.03	23.03	9.00
1# here means drug concentration in the blood of No. 1 rat.			

Table 23. Blood-drug concentration of berberine when the proportion of coptis, evodia and radix paeoniae alba is 6:0:0.			
Time(min)	1#(ng/ml)	2#(ng/ml)	3#(ng/ml)
5	50.71	89.28	73.73
15	137.45	137.89	128.50
30	271.01	254.36	287.59
60	460.44	532.30	512.00
120	195.38	185.55	245.00
180	123.46	152.54	158.03
240	94.79	112.32	109.04
360	31.44	58.61	54.02
480	22.09	35.25	43.78
1# here means drug concentration in the blood of No. 1 rat.			

Table 24. Blood-drug concentration of berberine when the proportion of coptis, evodia and radix paeoniae alba is 12:0:0.			
Time(min)	1#(ng/ml)	2#(ng/ml)	3#(ng/ml)
5	76.73	78.66	51.97
15	157.51	189.23	154.89
30	274.42	318.34	362.02
60	534.08	630.59	589.03
120	392.19	444.39	440.23
180	232.97	380.98	346.05
240	186.45	152.99	187.47
360	106.44	99.21	93.40
480	95.89	59.16	86.89
1# here means drug concentration in the blood of No. 1 rat.			

Blood-drug concentration of palmatine (Table 25-36)

Table 25. Blood-drug concentration of palmatine when the proportion of coptis, evodia and radix paeoniae alba is 3:1:3.			
Time(min)	1#(ng/ml)	2#(ng/ml)	3#(ng/ml)
5	8.90	10.21	4.38
15	10.34	21.22	7.44
30	31.08	36.91	24.33
60	22.10	12.29	12.80
120	15.74	5.42	9.24
180	12.37	11.49	9.89
240	5.89	10.44	8.77
360	4.39	7.65	7.05
480	2.90	5.28	5.92
1# here means drug concentration in the blood of No. 1 rat.			

Table 26. Blood-drug concentration of palmatine when the proportion of coptis, evodia and radix paeoniae alba is 3:2:6.			
Time(min)	1#(ng/ml)	2#(ng/ml)	3#(ng/ml)
5	3.90	5.29	3.10
15	5.84	6.90	11.27
30	13.54	14.08	29.18
60	19.78	44.89	55.13
120	41.49	22.60	23.91
180	30.59	13.52	14.90
240	24.54	15.46	10.52
360	13.26	11.46	9.79
480	7.68	9.49	10.22
1# here means drug concentration in the blood of No. 1 rat.			

Table 27. Blood-drug concentration of palmatine when the proportion of coptis, evodia and radix paeoniae alba is 3:6:12.			
Time(min)	1#(ng/ml)	2#(ng/ml)	3#(ng/ml)
5	7.61	8.90	12.25
15	20.68	11.83	26.31
30	69.74	44.22	46.75
60	46.48	43.17	38.70
120	33.04	23.46	34.62
180	20.45	19.00	22.17
240	21.83	17.15	13.10
360	11.65	10.35	18.93
480	12.80	6.28	4.68
1# here means drug concentration in the blood of No. 1 rat.			

Table 28. Blood-drug concentration of palmatine when the proportion of coptis, evodia and radix paeoniae alba is 6:1:6.			
Time(min)	1#(ng/ml)	2#(ng/ml)	3#(ng/ml)
5	38.96	51.33	66.60
15	109.57	117.62	86.89
30	88.96	91.93	85.86
60	72.41	97.85	70.25
120	62.02	82.03	71.63
180	64.08	70.50	58.55
240	41.65	38.69	46.95
360	25.22	44.83	35.56
480	11.68	10.62	24.70
1# here means drug concentration in the blood of No. 1 rat.			

Table 29. Blood-drug concentration of palmatine when the proportion of coptis, evodia and radix paeoniae alba is 6:2:12.			
Time(min)	1#(ng/ml)	2#(ng/ml)	3#(ng/ml)
5	87.62	63.69	71.91
15	108.00	98.89	117.73
30	153.14	178.99	146.51
60	111.28	131.39	117.88
120	129.71	99.91	89.18
180	101.58	101.43	75.12
240	68.08	41.37	58.19
360	43.78	53.74	39.39
480	45.89	41.60	33.12
1# here means drug concentration in the blood of No. 1 rat.			

Table 30. Blood-drug concentration of palmatine when the proportion of coptis, evodia and radix paeoniae alba is 6:6:3.			
Time(min)	1#(ng/ml)	2#(ng/ml)	3#(ng/ml)
5	47.39	76.34	94.29
15	81.31	101.63	58.23
30	116.86	89.11	60.86
60	129.37	87.30	85.09
120	136.18	105.60	122.35
180	102.19	86.46	104.73
240	65.99	62.69	80.18
360	76.77	59.36	61.76
480	52.67	55.75	40.57
1# here means drug concentration in the blood of No. 1 rat.			

Table 31. Blood-drug concentration of palmatine when the proportion of coptis, evodia and radix paeoniae alba is 12:1:12.			
Time(min)	1#(ng/ml)	2#(ng/ml)	3#(ng/ml)
5	32.51	23.37	22.09
15	10.74	25.74	59.41
30	17.83	29.91	61.49
60	39.97	42.97	79.29
120	78.16	93.28	112.55
180	154.81	186.66	219.13
240	144.13	120.04	191.70
360	130.46	89.92	112.07
480	71.87	55.47	83.38
1# here means drug concentration in the blood of No. 1 rat.			

Table 32. Blood-drug concentration of palmatine when the proportion of coptis, evodia and radix paeoniae alba is 12:2:3.			
Time(min)	1#(ng/ml)	2#(ng/ml)	3#(ng/ml)
5	35.60	25.40	46.51
15	51.27	19.79	38.62
30	128.34	74.10	95.35
60	148.55	146.92	131.95
120	294.41	242.77	296.02
180	238.37	258.10	262.60
240	150.34	134.18	159.88
360	109.84	141.01	111.47
480	83.05	111.47	93.49
1# here means drug concentration in the blood of No. 1 rat.			

Table 33. Blood-drug concentration of palmatine when the proportion of coptis, evodia and radix paeoniae alba is 12:6:6.			
Time(min)	1#(ng/ml)	2#(ng/ml)	3#(ng/ml)
5	57.74	69.67	35.78
15	86.79	66.44	41.25
30	125.01	118.71	123.62
60	175.93	213.75	262.17
120	123.20	119.46	155.59
180	152.20	74.19	104.07
240	85.24	57.60	82.53
360	57.25	45.77	55.02
480	47.19	56.47	76.01
1# here means drug concentration in the blood of No. 1 rat.			

Table 34. Blood-drug concentration of palmatine when the proportion of coptis, evodia and radix paeoniae alba is 3:0:0.			
Time(min)	1#(ng/ml)	2#(ng/ml)	3#(ng/ml)
5	3.63	3.51	3.98
15	7.37	7.28	8.79
30	19.46	12.13	12.29
60	54.14	53.67	46.36
120	27.82	28.68	25.57
180	17.35	16.00	20.04
240	13.23	12.34	14.70
360	5.28	5.63	2.86
480	2.41	2.87	3.53
1# here means drug concentration in the blood of No. 1 rat.			

Table 35. Blood-drug concentration of palmatine when the proportion of coptis, evodia and radix paeoniae alba is 6:0:0.			
Time(min)	1#(ng/ml)	2#(ng/ml)	3#(ng/ml)
5	20.33	15.87	21.16
15	55.49	42.60	37.24
30	85.44	66.08	54.41
60	132.34	120.62	114.85
120	79.49	85.05	37.46
180	40.84	46.94	46.64
240	15.05	26.51	24.64
360	10.29	15.38	25.05
480	8.76	6.44	10.85
1# here means drug concentration in the blood of No. 1 rat.			

Table 36. Blood-drug concentration of palmatine when the proportion of coptis, evodia and radix paeoniae alba is 12:0:0.			
Time(min)	1#(ng/ml)	2#(ng/ml)	3#(ng/ml)
5	25.38	29.18	16.47
15	58.93	33.36	29.05
30	136.85	91.75	73.20
60	206.89	188.06	167.00
120	143.81	158.71	143.91
180	71.66	131.47	118.09
240	63.92	92.92	84.19
360	40.77	32.82	30.56
480	35.02	19.16	27.45
1# here means drug concentration in the blood of No. 1 rat.			

Blood-drug concentration of evodiamine (Table 37-47)

Table 37. Blood-drug concentration of evodiamine when the proportion of coptis, evodia and radix paeoniae alba is 3:1:3.			
Time(min)	1#(ng/ml)	2#(ng/ml)	3#(ng/ml)
5	1.66	1.83	1.97
15	1.72	2.40	1.25
30	9.32	6.84	8.33
60	21.76	20.80	17.14
120	15.61	9.78	12.05
180	10.23	6.72	8.42
240	6.03	6.33	5.28
360	4.28	4.31	3.66
480	4.86	3.64	2.54
1# here means drug concentration in the blood of No. 1 rat.			

Table 38. Blood-drug concentration of evodiamine when the proportion of coptis, evodia and radix paeoniae alba is 3:2:6.			
Time(min)	1#(ng/ml)	2#(ng/ml)	3#(ng/ml)
5	6.43	4.65	3.72
15	13.34	9.80	16.59
30	26.37	24.43	31.33
60	22.63	32.03	18.78
120	23.77	23.45	11.26
180	21.07	19.95	13.04
240	14.68	13.42	10.54
360	9.17	9.69	5.85
480	5.45	4.20	2.54
1# here means drug concentration in the blood of No. 1 rat.			

Table 39. Blood-drug concentration of evodiamine when the proportion of coptis, evodia and radix paeoniae alba is 3:6:12.			
Time(min)	1#(ng/ml)	2#(ng/ml)	3#(ng/ml)
5	15.24	24.01	35.36
15	55.67	45.22	66.92
30	74.56	65.97	62.59
60	88.87	81.68	92.75
120	105.48	97.60	107.48
180	86.32	62.62	91.31
240	71.41	56.39	75.53
360	30.23	45.43	60.97
480	25.28	19.01	32.54
1# here means drug concentration in the blood of No. 1 rat.			

Table 40. Blood-drug concentration of evodiamine when the proportion of coptis, evodia and radix paeoniae alba is 6:2:12.			
Time(min)	1#(ng/ml)	2#(ng/ml)	3#(ng/ml)
5	6.47	8.09	7.84
15	22.07	16.99	25.03
30	21.80	19.27	20.67
60	27.41	25.83	31.42
120	40.72	31.21	51.06
180	32.56	35.24	48.69
240	20.68	25.42	32.54
360	22.20	16.90	12.76
480	13.06	12.10	13.43
1# here means drug concentration in the blood of No. 1 rat.			

Table 41. Blood-drug concentration of evodiamine when the proportion of coptis, evodia and radix paeoniae alba is 6:6:3.			
Time(min)	1#(ng/ml)	2#(ng/ml)	3#(ng/ml)
5	30.47	39.59	24.07
15	36.07	35.13	30.53
30	80.23	67.86	71.08
60	140.62	113.07	123.44
120	118.64	121.68	97.62
180	87.35	68.40	56.33
240	93.04	86.42	31.09
360	45.20	47.49	40.95
480	25.67	33.09	22.54
1# here means drug concentration in the blood of No. 1 rat.			

Table 42. Blood-drug concentration of evodiamine when the proportion of coptis, evodia and radix paeoniae alba is 12:1:12.			
Time(min)	1#(ng/ml)	2#(ng/ml)	3#(ng/ml)
5	1.82	1.96	2.08
15	3.63	5.83	1.88
30	7.61	6.10	5.79
60	18.32	17.22	16.67
120	7.24	14.45	14.92
180	3.07	4.32	12.65
240	4.13	6.65	5.65
360	2.36	2.65	3.65
480	3.65	4.18	3.01
1# here means drug concentration in the blood of No. 1 rat.			

Table 43. Blood-drug concentration of evodiamine when the proportion of coptis, evodia and radix paeoniae alba is 12:2:3.			
Time(min)	1#(ng/ml)	2#(ng/ml)	3#(ng/ml)
5	8.54	11.33	16.94
15	8.90	18.15	18.00
30	16.63	17.95	26.66
60	29.46	39.70	26.48
120	57.20	78.83	62.16
180	45.60	50.24	35.61
240	34.21	26.02	34.00
360	24.48	33.39	22.65
480	12.65	17.65	15.65
1# here means drug concentration in the blood of No. 1 rat.			

Table 44. Blood-drug concentration of evodiamine when the proportion of coptis, evodia and radix paeoniae alba is 12:6:6.			
Time(min)	1#(ng/ml)	2#(ng/ml)	3#(ng/ml)
5	23.34	38.12	18.05
15	42.78	36.91	31.22
30	67.68	55.80	74.31
60	89.82	77.56	64.77
120	57.07	44.54	46.89
180	55.22	54.41	27.83
240	32.65	42.65	22.65
360	17.41	20.50	17.13
480	19.54	12.65	15.92
1# here means drug concentration in the blood of No. 1 rat.			

Table 45. Blood-drug concentration of evodiamine when the proportion of coptis, evodia and radix paeoniae alba is 3:0:0.			
Time(min)	1#(ng/ml)	2#(ng/ml)	3#(ng/ml)
5	3.08	3.44	2.57
15	7.44	6.56	4.19
30	11.25	11.08	12.06
60	7.11	9.23	9.32
120	6.77	6.14	6.34
180	5.41	5.08	5.12
240	3.07	2.51	4.11
360	1.03	1.81	3.06
480	0.98	1.40	1.81
1# here means drug concentration in the blood of No. 1 rat.			

Table 46. Blood-drug concentration of evodiamine when the proportion of coptis, evodia and radix paeoniae alba is 6:0:0.			
Time(min)	1#(ng/ml)	2#(ng/ml)	3#(ng/ml)
5	6.10	5.21	7.21
15	14.73	17.03	16.27
30	23.27	33.46	25.92
60	16.08	19.37	19.01
120	11.40	14.90	15.37
180	8.71	8.28	11.78
240	5.83	5.06	8.14
360	2.05	2.45	4.01
480	1.95	1.58	2.45
1# here means drug concentration in the blood of No. 1 rat.			

Table 47. Blood-drug concentration of evodiamine when the proportion of coptis, evodia and radix paeoniae alba is 12:0:0.			
Time(min)	1#(ng/ml)	2#(ng/ml)	3#(ng/ml)
5	12.31	15.64	21.64
15	34.18	39.08	48.80
30	56.20	64.32	66.76
60	38.32	59.12	42.04
120	21.00	44.69	38.12
180	18.23	32.34	34.69
240	13.25	24.34	22.83
360	7.00	19.13	15.19
480	5.23	8.71	7.34
1# here means drug concentration in the blood of No. 1 rat.			

Blood-drug concentration of rutecarpine (Table 48-59)

Table 48. Blood-drug concentration of rutecarpine when the proportion of coptis, evodia and radix paeoniae alba is 3:1:3.

Time(min)	1#(ng/ml)	2#(ng/ml)	3#(ng/ml)
5	8.55	3.92	4.29
15	10.26	15.67	7.03
30	11.05	21.93	12.57
60	32.30	27.93	18.34
120	14.58	17.24	13.33
180	16.86	10.11	15.37
240	7.36	16.85	11.82
360	6.71	13.87	6.50
480	6.36	12.07	5.24

1# here means drug concentration in the blood of No. 1 rat.

Table 49. Blood-drug concentration of rutecarpine when the proportion of coptis, evodia and radix paeoniae alba is 3:2:6.

Time(min)	1#(ng/ml)	2#(ng/ml)	3#(ng/ml)
5	10.56	11.52	7.10
15	23.23	27.90	17.29
30	47.06	51.01	43.88
60	31.17	38.03	46.69
120	35.92	31.53	35.86
180	40.66	34.04	31.03
240	36.03	28.38	27.77
360	21.64	23.78	13.57
480	11.97	20.11	11.99

1# here means drug concentration in the blood of No. 1 rat.

Table 50. Blood-drug concentration of rutecarpine when the proportion of coptis, evodia and radix paeoniae alba is 3:6:12.

Time(min)	1#(ng/ml)	2#(ng/ml)	3#(ng/ml)
5	26.08	35.48	23.24
15	52.29	72.57	60.47
30	105.39	145.81	140.99
60	89.94	57.13	118.56
120	46.66	63.10	53.66
180	64.09	41.19	37.21
240	33.72	32.51	22.19
360	28.66	41.01	24.64
480	24.93	28.61	33.19

1# here means drug concentration in the blood of No. 1 rat.

Table 51. Blood-drug concentration of rutecarpine when the proportion of coptis, evodia and radix paeoniae alba is 6:1:6.

Time(min)	1#(ng/ml)	2#(ng/ml)	3#(ng/ml)
5	4.73	5.54	9.22
15	16.78	25.73	19.63
30	13.58	16.98	10.48
60	14.53	16.19	11.54
120	12.27	10.12	7.01
180	8.02	8.02	9.35
240	6.21	8.93	7.00
360	5.41	3.73	5.34
480	4.28	4.74	6.77

1# here means drug concentration in the blood of No. 1 rat.

Table 52. Blood-drug concentration of rutecarpine when the proportion of coptis, evodia and radix paeoniae alba is 6:2:12.			
Time(min)	1#(ng/ml)	2#(ng/ml)	3#(ng/ml)
5	15.60	23.50	16.42
15	63.36	48.64	37.94
30	47.13	31.83	26.33
60	36.36	19.17	25.81
120	26.42	25.53	14.22
180	15.61	19.99	15.54
240	11.75	13.98	13.05
360	11.82	11.35	11.94
480	7.93	10.29	7.75
1# here means drug concentration in the blood of No. 1 rat.			

Table 53. Blood-drug concentration of rutecarpine when the proportion of coptis, evodia and radix paeoniae alba is 6:6:3.			
Time(min)	1#(ng/ml)	2#(ng/ml)	3#(ng/ml)
5	33.41	37.29	41.00
15	68.23	45.42	61.40
30	90.21	84.21	88.97
60	105.84	129.48	125.21
120	115.02	90.08	87.67
180	68.67	59.73	62.90
240	50.57	45.47	52.38
360	33.97	53.98	42.22
480	45.81	32.87	26.45
1# here means drug concentration in the blood of No. 1 rat.			

Table 54. Blood-drug concentration of rutecarpine when the proportion of coptis, evodia and radix paeoniae alba is 12:1:2.			
Time(min)	1#(ng/ml)	2#(ng/ml)	3#(ng/ml)
5	8.29	10.40	7.92
15	13.75	9.51	13.14
30	27.96	12.12	17.93
60	20.81	15.22	14.95
120	15.47	10.42	6.38
180	12.82	11.61	8.75
240	6.78	9.76	8.97
360	6.91	5.62	6.84
480	5.28	3.75	3.25
1# here means drug concentration in the blood of No. 1 rat.			

Table 55. Blood-drug concentration of rutecarpine when the proportion of coptis, evodia and radix paeoniae alba is 12:2:3.			
Time(min)	1#(ng/ml)	2#(ng/ml)	3#(ng/ml)
5	12.91	14.49	14.30
15	18.44	30.81	21.95
30	16.82	31.07	15.03
60	21.90	37.46	23.45
120	36.51	42.80	35.68
180	21.18	45.39	17.66
240	22.07	24.09	21.10
360	16.12	19.29	13.75
480	13.39	13.75	17.83
1# here means drug concentration in the blood of No. 1 rat.			

Table 56. Blood-drug concentration of rutecarpine when the proportion of coptis, evodia and radix paeoniae alba is 12:6:6.			
Time(min)	1#(ng/ml)	2#(ng/ml)	3#(ng/ml)
5	33.51	28.80	25.37
15	35.80	34.61	36.97
30	68.23	70.37	74.31
60	92.63	104.21	84.12
120	68.85	91.72	69.06
180	53.75	43.02	56.02
240	36.38	49.20	43.42
360	27.47	33.71	17.93
480	23.90	16.21	11.87
1# here means drug concentration in the blood of No. 1 rat.			

Table 57. Blood-drug concentration of rutecarpine when the proportion of coptis, evodia and radix paeoniae alba is 0:1:0.			
Time(min)	1#(ng/ml)	2#(ng/ml)	3#(ng/ml)
5	4.76	5.34	4.50
15	7.25	7.74	6.75
30	11.51	11.78	15.56
60	5.36	9.13	9.81
120	4.08	5.70	7.25
180	3.54	5.23	4.51
240	3.33	5.63	3.32
360	1.82	1.82	2.21
480	1.32	1.46	2.11
1# here means drug concentration in the blood of No. 1 rat.			

Table 58. Blood-drug concentration of rutecarpine when the proportion of coptis, evodia and radix paeoniae alba is 0:2:0.			
Time(min)	1#(ng/ml)	2#(ng/ml)	3#(ng/ml)
5	6.77	5.93	5.51
15	13.17	19.75	10.33
30	32.35	30.00	27.48
60	19.14	11.93	18.13
120	14.13	7.80	14.60
180	8.80	4.29	9.60
240	6.47	2.67	5.72
360	4.31	2.00	4.46
480	3.11	1.43	2.23
1# here means drug concentration in the blood of No. 1 rat.			

Table 59. Blood-drug concentration of rutecarpine when the proportion of coptis, evodia and radix paeoniae alba is 0:6:0.			
Time(min)	1#(ng/ml)	2#(ng/ml)	3#(ng/ml)
5	20.30	23.79	16.52
15	49.51	59.25	30.98
30	77.04	69.99	82.43
60	57.41	65.80	54.39
120	42.39	43.39	28.80
180	36.40	32.88	17.15
240	29.42	28.02	13.37
360	16.62	13.49	11.78
480	9.33	6.97	4.86
1# here means drug concentration in the blood of No. 1 rat.			

Supplementary RESULTS

Basic statistics of the original data

Range and variance analysis According to the value of range, the effect of every element to berberine is B > A > C. Through the result of variance analysis, different level of coptis and evodia fructus have significant difference ($P < 0.01$), F means the contribution of the corresponding component. For absorption fraction of berberine, the contribution of tree components is B > A > C. According to the result of range and variance analysis, the optimal compatibility proportion is 12:6:6 (Table 60).

Table 60. Analysis of variance of berberine.				
Component	Quadratic sum	Mean square error	F	P
Coptis(A)	0.097	0.049	12.098	0.000
Evodia fructus(B)	0.174	0.087	1.602	0.000
Radix paeoniae alba(C)	0.011	0.006	1.368	0.280
Blank line*	0.037	0.019	4.653	0.024
Error	0.072	0.004		
*Blank line here means use perfusate without adding any drug.				

According to Table 61, we draw a similar conclusion, and the optimal compatibility proportion is 12:6:6.

Table 61. Analysis of variance of palmatine.				
Component	Quadratic sum	Mean square error	F	P
Coptis(A)	0.149	0.074	11.088	0.001
Evodia fructus(B)	0.206	0.103	15.361	0.000
Radix paeoniae alba(C)	0.011	0.006	0.827	0.453
Blank line*	0.046	0.023	3.424	0.055
Error	0.121	0.007		

*Blank line here means use perfusate without adding any drug.

Based on the above analysis, the effect of single herb in Wuji pill for uptake of berberine and palmatine is shown in Table 61. Contribution denotes the effect degree that each component does to the concentration of corresponding ingredient, the more contribution, the more effect degree. The relation between them can be promoting or suppressing, we use '-' to denote suppressing and '+' to denote promoting. Therefore, we can sort the value of influence factor by sorting contribution. From the statistical result, we can conclude that coptis play a leading role to concentration of Ber (berberine), Pal (palmatine), Pae (paeoniflorin). There is a positive correlation between coptis and Ber, Pal.

Table 62. Effect of individual herb on concentration of five ingredients and the optimal compatibility in Wuji pill.

	Contribution type				Optimal compatibility proportion	
Ingredient	A*	B*	C*	Contribution		
Ber	+			A > B > C	A-B-C	12 : 6 : 6
Pal	+			A > B > C	A-B-C	12 : 6 : 6
Evo	+		-	B > A > C	A-B-C	12 : 6 : 6
Rut		+		B > C > A	A-B-C	12 : 6 : 6
Pae				A > B > C	A-B-C	12 : 1 : 6

*A denotes coptis; B denotes evodia fructus; C denotes radix paeoniae alba.

REFERENCES

1. Pang, K. S., Cherry, W. F. & Ulm, E. H. Disposition of enalapril in the perfused rat intestine-liver preparation: absorption, metabolism and first-pass effect. *J. Pharmacol. Exper. Ther.* **233,** 788-795 (1985).
